# Unraveling the Mechanism of PEG-Enhanced Ultrasound-Triggered Release from Liposomes

**DOI:** 10.1021/acs.langmuir.6c03550

**Published:** 2026-09-03

**Authors:** Ignasi Simon, Farzaneh Gholamianpour, Lassi V. Tiihonen, Simon Vliek, Amber Renkema, Teun L.A. Janssen, Coen Fransen, Steven R. Parnell, Riccardo Alessandri, Remco Hartkamp, Wim G. Bouwman, Tiago L. da Costa, Alina Y. Rwei

**Affiliations:** † Department of Chemical Engineering, 2860 Delft University of Technology, Delft 2629 HZ, The Netherlands; ‡ Department of Microelectronics, Delft University of Technology, Delft 2628 CD, The Netherlands; § Department for Radiation Science and Technology, 2860 Delft University of Technology, Delft 2629 JB, The Netherlands; ∥ ISIS Neutron and Muon Source, 120797 Rutherford Appleton Laboratory, Chilton, Oxon OX11 0QX U.K.; ⊥ Department of Chemical Engineering, 418666 KU Leuven, Leuven 3001, Belgium; # Department of Process & Energy, Delft University of Technology, Delft 2628 CB, The Netherlands

## Abstract

Ultrasound-responsive liposomes represent a promising strategy for the targeted delivery of therapeutic agents to deep tissues, combining the clinically validated biocompatibility of liposomes with precise spatiotemporal control via ultrasound. While the incorporation of polyethylene glycol-conjugated lipids into liposomal formulations has long been known to enhance ultrasound-triggered release, the mechanistic basis of this enhancement remains poorly understood, hindering the rational design of formulations with enhanced ultrasound-triggered release. To address this, we investigated the proposed hypotheses in the literature, including the influence of the packing parameter, differences in thermal, mechanical, and fluidity properties, as well as structural changes such as micelle formation or bilayer thinning for stealth liposomes containing polyethylene glycol, polycarboxybetaine, or polysarcosine. Our results indicate that PEG-enhanced ultrasound-triggered release cannot be attributed to changes in bulk bilayer properties, including the packing parameter, membrane mechanics, thermal behavior, or fluidity. Moreover, the membrane structure remained unchanged after insonation, with no evidence of micelle ejection or membrane thinning. Instead, experiments and computational simulations suggest that polyethylene glycol-conjugated lipopolymers facilitate sonoporation by rearranging into micelle-like structures at the periphery of ultrasound-induced pores in the lipid bilayer, enabled by their low critical micelle concentration, thereby enhancing the release through these transient openings. Finally, we discuss how these insights could be extended beyond polyethylene glycol lipopolymers to guide the design of liposomes with enhanced ultrasound-triggered release, by shifting optimization from bulk bilayer properties to a sonoporation-oriented framework that integrates computational screening, AI-enabled formulation design, and ultrasound waveform considerations.

## Graphical abstract

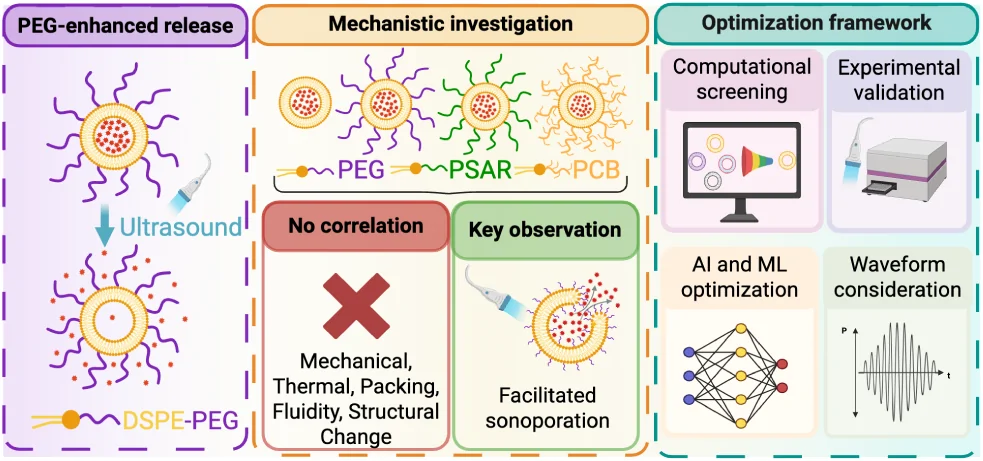

## Introduction

Nanocarriers can overcome the limitations of free active pharmaceutical ingredients by enhancing their solubility and stability, extending circulation time to improve therapeutic profiles, and modulating biodistribution to minimize systemic exposure and improve safety.^1,2^ Among the myriad of nanocarriers engineered to date, lipid-based nanoparticles have received substantial academic and industrial attention for the delivery of a wide range of therapeutic cargoes, including small molecules and nucleic acids.^3^ While lipid nanoparticles are particularly suited for encapsulating genetic material, liposomes are the preferred choice for delivering small-molecule drugs.^4^ Nevertheless, inadequate payload release at the target site in clinically approved liposomal formulations, such as Doxil and Myocet, continues to limit therapeutic efficacy.^5^ To overcome this challenge, liposomes capable of releasing their therapeutic cargo in response to endogenous and external stimuli have been investigated.^6^

Ultrasound (US) stands out as an external triggering technology due to its noninvasiveness, deep-tissue penetration (attenuation of ∼0.7 dB/cm/MHz in soft tissue),^7^ high spatial precision (wavelengths in the submillimeter range at typical MHz US frequencies), proven clinical safety,^8^ and growing momentum from established and emerging therapeutic uses, including neuromodulation,^9^ histotripsy,^10^ and enhanced debris clearance.^11^ A variety of US-responsive drug-delivery carriers have been developed, including microbubbles and phase-changing nanodroplets.^12^ Among them, nanosized liposomes capable of releasing their cargo upon US insonation without the use of perfluorocarbons or sonosensitizers—here referred to as US-responsive liposomes—offer significant promise due to their high biocompatibility and efficient encapsulation of a range of therapeutic cargoes.^13,14^ However, to improve the therapeutic potential of US-responsive liposomes and facilitate their clinical translation, formulations must be designed to achieve rapid and efficient cargo release upon US triggering.

To improve the US-triggered release efficiency of liposomes, several groups have systematically examined the effects of lipid composition on US-triggered release efficiency,^14^ including cholesterol content,^15^ lipid headgroup type,^16^ targeting moieties,^17,18^ incorporation of lysolipids,^19^ and aqueous core composition.^20^ Notably, all previous liposomal formulations included 1,2-distearoyl-*sn*-glycero-3-phosphoethanolamine-N-[methoxy­(polyethylene glycol)-2000] (DSPE-PEG2000).^21^ Despite being identified as a key lipid for enhanced release efficiency in early studies,^13,21–23^ the mechanism by which PEGylation substantially enhances US-triggered release has long remained poorly understood. Hypothesized mechanisms include the absorption of ultrasonic energy by the highly hydrated PEG headgroups,^13^ a lower packing parameter (PP),^15,19,24^ changes in membrane fluidity or mechanical properties,^16,22^ differences in transition temperature (*T*_m_),^16^ and PEG lipopolymer ejection leading to micelle formation upon sonication.^18,22^ However, no consensus has been reached on the mechanistic role of DSPE-PEG in improving the efficiency of US-triggered release. Such a limited understanding hinders the design of more effective US-responsive liposomal formulations, which is critical for successful clinical translation and improved therapeutic outcomes.

To address this knowledge gap, we investigated US-triggered release dynamics across bare liposomes, stealth PEGylated liposomes, and liposomes functionalized with alternative hydrophilic polymers, namely polysarcosine (PSAR) and polycarboxybetaine (PCB). PSAR and zwitterionic PCB were selected because they have attracted attention as potential stealth alternatives to PEG for both liposomes and lipid nanoparticles,^25,26^ addressing limitations such as the anti-PEG immune response associated with repeated dosing with PEGylated formulations.^27^ We initially hypothesized that if the low PP or the high hydration of the polymer was the primary factor underlying PEG’s enhancement in US-triggered release, all lipopolymers would similarly enhance US-triggered release compared to bare liposomes. Contrary to our expectations, only the PEGylated formulation displayed significantly higher release upon US insonation, indicating a unique role of PEG lipopolymers in enhancing release efficiency.

To elucidate this PEG-driven enhancement, we comprehensively characterized all formulations by assessing release kinetics, cargo retention, and size stability; mechanical properties via atomic force microscopy (AFM) nanoindentation, bilayer fluidity via fluorescence polarization (FP), phase transition shifts using differential scanning calorimetry (DSC), lipid bilayer structure analysis with small-angle neutron scattering (SANS), and critical micelle concentration (CMC) characterization. The PEG-enhanced US-triggered release was found to be independent of bulk bilayer properties, including lipid packing parameter, membrane mechanics, thermal behavior, and fluidity. Moreover, no detectable structural alterations after insonation, such as micelle ejection or bilayer thinning, were observed. Instead, experiments and coarse-grained molecular dynamics simulations (CG-MD) suggest that PEGylated lipids—distinguished by a higher micellization tendency (i.e., a lower CMC than PSAR and PCB lipopolymers)—promote pressure-induced sonoporation by reorganizing into micelle-like assemblies at US-generated transient pores, ultimately facilitating enhanced US-triggered release through these defects. Finally, we discuss how these mechanistic insights could guide the design of more efficient US-responsive liposomes beyond the PEG-driven enhancement. Rather than focusing solely on bulk bilayer properties, the proposed approach centers on sonoporation as the primary rationale for formulation optimization, integrating computational screening, artificial intelligence (AI)-enabled design strategies, and acoustic waveform considerations.

## Results and Discussion

### US-Triggered Release from Liposomes is Enhanced by DSPE-PEG But Not by DSPE-PSAR or DSPE-PCB

Previous studies argued that PEG enhances US-triggered release from liposomes through its low PP or due to absorption of acoustic energy associated with a thick, highly hydrated polymer corona.^15,19^ To examine these hypotheses, we investigated liposomes functionalized with alternative hydrophilic polymers commonly utilized for stealthing, specifically PSAR and PCB. Similar to PEG, these hydrophilic polymers form a hydrated corona around the liposomes and, due to their bulky polymer headgroups, confer a low PP (i.e., well below 1), in contrast to ∼1 for bilayer-forming phospholipids such as 1,2-distearoyl-*sn*-glycero-3-phosphocholine (DSPC).^28,29^ We therefore reasoned that if the low PP or the US energy absorption were the dominant contributors to the enhanced release of PEGylated formulations, lipopolymers with similarly low PP and comparably hydrated polymeric layers would exhibit analogous US-triggered release profiles. Accordingly, liposomes encapsulating self-quenched calcein were prepared with 5 mol % DSPE-polymer conjugates (PEG, PCB, or PSAR), along with a nonstealth formulation, using 48 mol % DSPC, 22 mol % 1,2-dioleoyl-*sn*-glycero-3-phosphocholine (DOPC), and 25 mol % cholesterol. All formulations exhibited comparable hydrodynamic diameters (Figure S1A) with a low (<0.1) PDI (Figure S1B), neutral to slightly negative ζ-potentials (Figure S1C), and similar cargo retention at storage conditions (4 °C) (Figure S1D).

Upon US insonation (3 W·cm^–2^, 1 MHz, 50% duty cycle, 1 Hz pulse repetition frequency), PEGylated liposomes displayed significantly higher release efficiency (∼7x after 10 min insonation), as measured by calcein dequenching, than the nonstealth control across all time points (Figure 1A). In contrast, PCB- and PSAR-functionalized liposomes showed minimal release (<10% after 10 min under the same US conditions), similar to that of bare liposomes (Figure 1A). For PEGylated formulations, the extent of release increased with PEG molar fraction, consistent with previous reports (Figure S2A).^22^ Yet neither PCB nor PSAR promoted release across the range of molar fractions examined (Figure S2B and S2C). Collectively, these results indicate that PEGylation—rather than stealthing with alternative hydrophilic polymers such as PCB and PSAR—is the key determinant of US responsiveness in liposomes. The lack of comparable enhancement in PCB- and PSAR-coated liposomes further suggests that a reduced PP of the lipopolymer, or the presence of a highly hydrated polymer corona alone, is insufficient to account for enhanced US-triggered release.^15,19,24^ Accordingly, PSAR- and PCB-functionalized liposomes appear poorly suited for applications requiring efficient US-triggered cargo release, despite their promise in addressing anti-PEG immune responses associated with repeated dosing of PEGylated formulations.^25–27^

**Figure 1:**
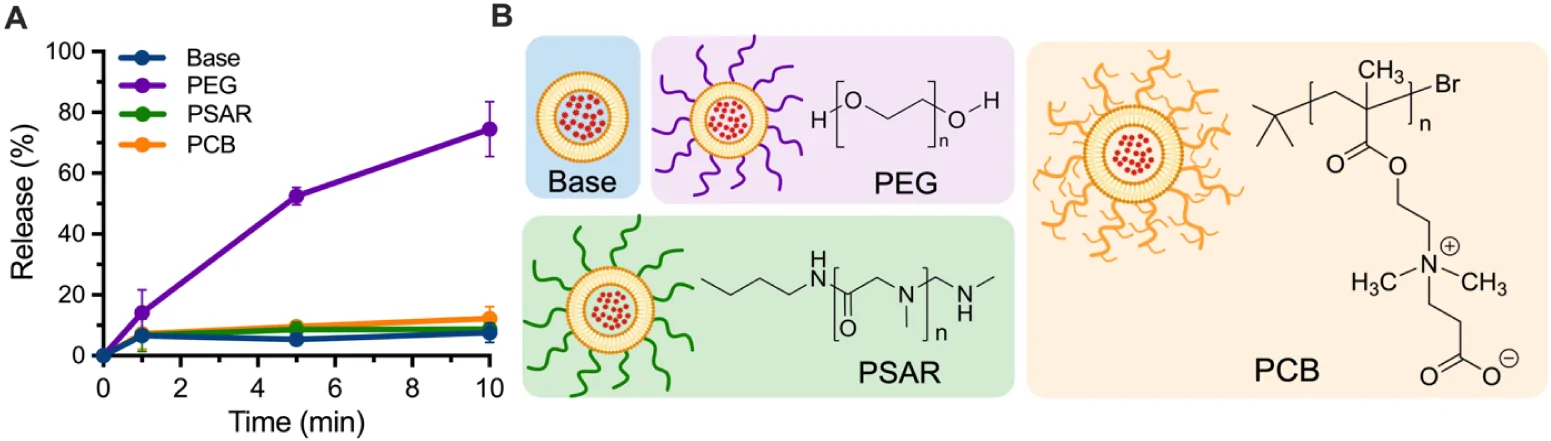
DSPE-PEG-driven enhanced US-triggered release from liposomes. (A) Cumulative release profiles under US exposure (3 W·cm^–2^ intensity, 1 MHz frequency, 50% duty cycle, 1 Hz pulse repetition frequency) as a function of time for bare, PEG-, PSAR-, and PCB-coated liposomes, showing that significant enhancement in US-triggered release occurs only for PEGylated formulations (*n* = 3). (B) Schematics of each liposomal formulation, illustrating the absence or presence of a stealth polymer layer, alongside the corresponding chemical structures of PEG, PSAR, and PCB. Although not depicted for clarity, the polymer chains are also present in the inner leaflets of the bilayer due to the extrusion-based preparation method. Schematics in (B) created with Biorender.com.

### Mechanical and Thermal Properties Do Not Correlate with DSPE-PEG-Enhanced Release Profiles

US can induce both thermal and mechanical effects on biological tissues, leading to membrane disruption, increased permeability, or structural rearrangements of cell bilayers.^30^ Similar bioeffects can act on artificial lipid bilayers,^31^ including those of liposomes. Therefore, one possible hypothesis is that PEGylation may modify bulk liposome properties—including fluidity, stiffness, or thermal properties—in a manner that enhances sensitivity to these US-induced bioeffects, while PCB and PSAR may not. Such properties are well known to be tunable through liposomal formulation engineering,^32,33^ and modulating them can have broad consequences, ranging from cargo retention and release kinetics to pharmacokinetics and biodistribution.^34^ Indeed, changes in membrane fluidity, mechanical properties,^16,22^ and *T*_m_^16^ have been proposed as determinants of enhanced US-triggered release from US-responsive liposomes. Motivated by the markedly distinct release profiles observed among PEG-, PCB-, and PSAR-coated liposomes and the nonstealth base formulation (Figure 1A), we sought to characterize their mechanical and thermal properties to identify potential relationships with the previously observed US-triggered release behavior.

As a mechanical pressure wave, US propagation and its associated effects are influenced by the medium’s mechanical and acoustic properties. Accordingly, variations in liposomal membrane mechanical properties could, in principle, modulate US-induced bilayer deformation by the pressure wave, thereby influencing release profiles. To evaluate whether differences in mechanical properties underlie variations in US-triggered release efficiency, AFM nanoindentation was used to quantify the stiffness of each formulation (Figure 2A).^35,36^ As AFM nanoindentation and ultrasound represent fundamentally different mechanical stimuli, and the liposomes are placed under distinct environments during measurements (i.e., adsorbed during AFM versus freely dispersed in ultrasound exposure), the measurements were intended solely for comparison of stiffness across formulations, rather than to replicate the mechanical effects of an acoustic pressure wave on the liposome membrane. To this end, measurements were performed in PBS (i.e., liquid AFM) within the elastic regime, where small indentation depths minimize substrate contributions to the measured response, and under identical substrate conditions across all formulations, ensuring direct comparability of the stiffness values. The PEGylated liposome, which had the highest release, showed a stiffness of 74.96 ± 2.10 pN/nm (mean ± standard error), higher than the PCB (43.71 ± 1.83 pN/nm) and base formulations (54.98 ± 2.51 pN/nm), but statistically indistinguishable from the PSAR formulation (75.45 ± 2.50 pN/nm) (Figure 2B). Despite the observed statistical differences in stiffness, there was no clear correlation between this property and release efficiency, suggesting that bilayer stiffness alone does not dictate the PEG-mediated enhancement in US responsiveness.

**Figure 2:**
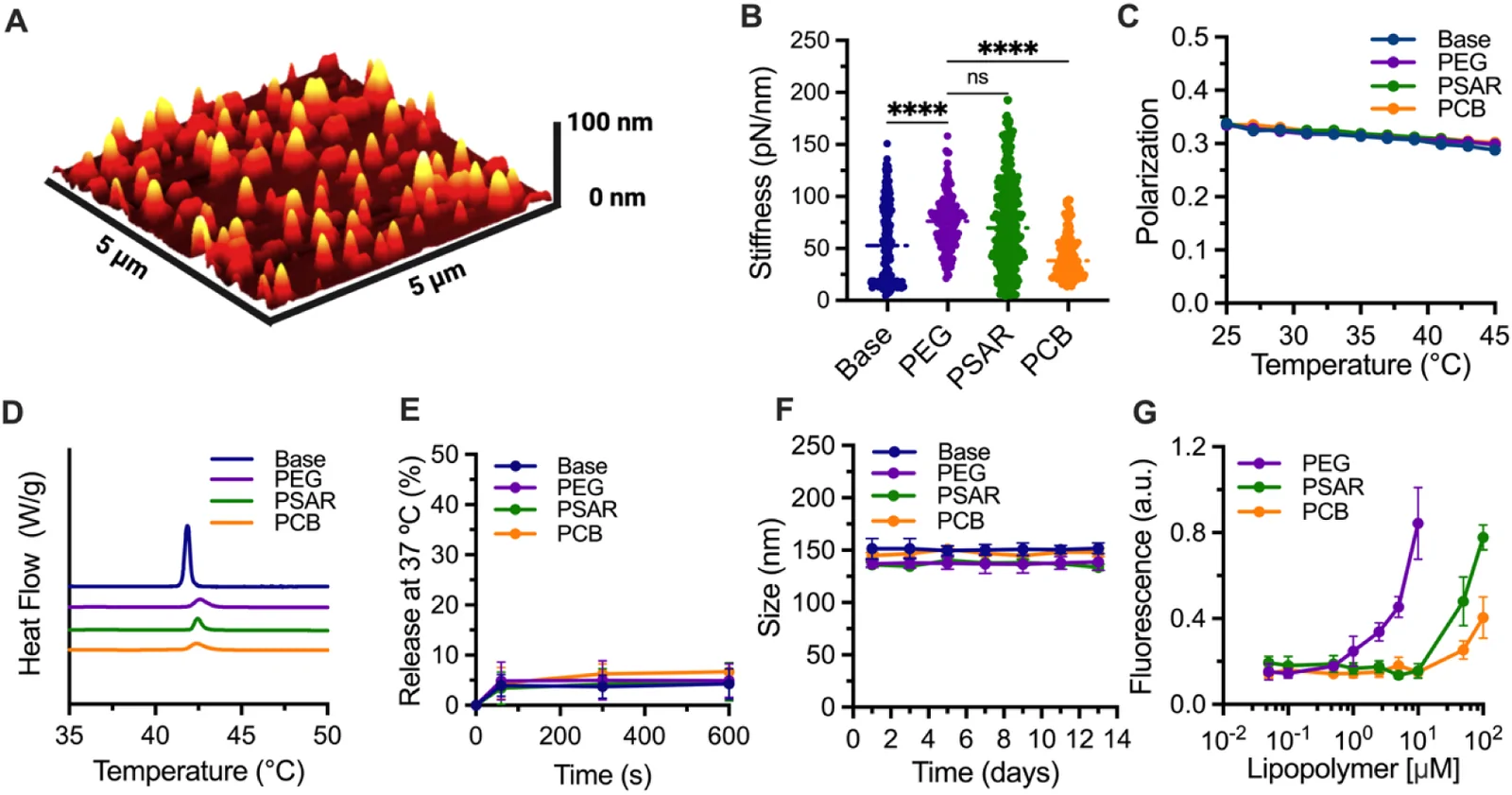
Characterization of liposome mechanical, thermal, fluidity, and stability properties, and lipopolymer CMC. (A) Representative three-dimensional AFM image of liposomes prior to nanoindentation. (B) AFM nanoindentation measurements of liposome stiffness, showing no correlation between mechanical rigidity and the enhanced US-triggered release observed for PEGylated formulations (*n* > 150 curves analyzed per group). ****p < 0.0005. (C) Fluorescence polarization measurements as a function of temperature (*n* = 2), with comparable polarization and therefore fluidity across all formulations. (D) DSC thermograms of bare and polymer-modified liposomes, showing similar phase transition temperature, *T*_m_, with equal broadening and peak reduction in stealth formulations. (E) Cargo retention stability at 37 °C (*n* = 3) and (F) hydrodynamic size stability at 4 °C (*n* = 3), confirming equivalent cargo retention and structural integrity across formulations. (G) CMC measurements of lipopolymers (*n* = 3), revealing a substantially lower CMC for PEG compared to PSAR and PCB.

Assessing fluidity is relevant in the context of US-triggered release, as more fluid membranes with higher lipid mobility could, in principle, be more susceptible to deformation under pressure-wave perturbations. Membrane fluidity was evaluated by fluorescence anisotropy, a technique commonly employed to measure lipid chain mobility and the dynamic behavior of lipid bilayers.^37^ Polarization decreased with increasing temperature across all formulations (Figure 2C), consistent with increased membrane fluidity driven by enhanced lipid chain mobility at elevated temperatures. This trend aligns with previous studies showing that the polarization of 1,6-diphenyl-1,3,5-hexatriene (DPH) in liposome membranes decreases with increasing temperature, indicating increased membrane fluidity.^38^ More importantly, all formulations displayed equal fluidity across temperatures (Figure 2C). In other words, there was no correlation between fluidity and US-triggered release, aligning with the expectation that stealth polymers, being surface-bound, do not dominantly alter intrinsic bilayer lipid dynamics. While we do not exclude the possibility that membrane fluidity may influence US responsiveness in systems where bilayer composition is directly modified—for example, via shorter acyl chain lipids or variations in lipid unsaturation content—the significantly enhanced US-triggered release by PEGylated lipids observed here could not be attributed to PEGylation-induced changes in membrane fluidity.

Temperature-responsive liposomes have been extensively developed to enable triggered drug release in response to mild hyperthermia, with clinically translated examples such as ThermoDox demonstrating that controllable, small increases in temperature near physiological ranges can substantially enhance membrane permeability and facilitate cargo release.^39^ US can induce localized heating alongside mechanical stresses, thereby raising the possibility that elevated temperatures contribute to the enhanced release observed in PEGylated liposomes. In this context, variations in *T*_m_ have been proposed as a primary factor governing US-triggered release in liposomes with different headgroups.^16^ To assess this, we first evaluated cargo retention stability at physiological temperature. All formulations displayed similar cargo entrapment stability at 37 °C (Figure 2E), the temperature reached within 10 min of insonation starting at room temperature (Figure S2D), as well as equal colloidal stability (Figure 2F) and cargo entrapment stability (Figure S1D) under storage conditions (4 °C). Furthermore, PEGylation did not induce markedly distinct shifts in transition temperature or phase behavior that were not similarly observed with DSPE-PCB or DSPE-PSAR (Figure 2D). In all cases, addition of DSPE-based lipopolymers resulted in a modest increase in *T*_m_ and peak broadening relative to the base formulation (Figure 2D). This is attributable to the incorporation of the high-*T*_m_ DSPE anchor, rather than to polymer-specific interactions, since PEG, PCB, and PSAR are all grafted onto the membrane. Thus, any effects on lipid phase behavior are predominantly governed by the insertion of DSPE itself rather than the identity of the polymer chain. Overall, the increase in US-triggered release efficiency for PEGylated formulations could not be attributed to changes in transition temperature or cargo retention stability at US-induced temperature increase.

### DSPE-PEG Does Not Eject as Micelles But Forms Micelle-Like Structures in US-Induced Pores

Collectively, the previous findings suggest that changes in bulk liposome properties, such as membrane fluidity, thermal stability, or mechanical stiffness, do not dictate the PEG-mediated enhancement of US-triggered release. Instead, such PEG-driven enhancement may be dominated by localized, transient events occurring during US insonation. In this context, PEG lipopolymers have been hypothesized to be ejected from the bilayer and to form micelles upon sonication, with such discrete, nonreversible events facilitating cargo release.^18,22^ This is supported by the intrinsic tendency of PEG lipids to self-assemble into micellar structures compared to conventional bilayer-forming lipids such as DSPC. To investigate this tendency, we measured the CMC of each lipopolymer, which reflects the thermodynamic propensity for micelle structure formation.^40,41^ CMCs were measured using the DPH probe method, previously validated for DSPE-PEG micelles with CMC values in the submicromolar to low-micromolar range.^42^ DSPE-PEG exhibited a CMC of ∼0.5–1 μM (Figure 2G), consistent with previous reports,^42,43^ one to 2 orders of magnitude lower than DSPE-PCB and DSPE-PSAR (∼10–50 μM) (Figure 2G). We note that the measured values approach the detection limit of the DPH fluorescence method, where factors such as variability in DPH partitioning or residual organic solvent from the stock solution can reduce the signal-to-noise ratio.^44^ To mitigate this, the CMC was determined from multiple independent measurements, focusing on consistent inflection behavior rather than individual data points. The resulting values are in good agreement with previous reports, as mentioned previously. Although these conditions introduce an inherent uncertainty margin, this is not expected to affect the order of magnitude of the reported CMC values. Thus, the markedly lower CMC reflects a strong thermodynamic drive for PEG lipids to organize into micelle-like structures. We hypothesized that this propensity could lead to micelle ejection upon insonation or the formation of transient micelle-like structures at US-induced pores, and determining which mechanism dominates is critical to understanding PEG-driven US responsiveness.

Size measurements were performed before and after insonation to investigate whether micelle formation occurred upon DSPE-PEG ejection, as well as to assess the possibility of complete liposome rupture following US insonation. Across all formulations, including PEG-coated liposomes exhibiting high release, dynamic light scattering (DLS) profiles consistently revealed a single, monodisperse population with no significant changes in mean diameter (Figure S3A–S3D) or PDI (Figure S3E–S3H) following US exposure. US-triggered release thus occurred without vesicle rupture, in agreement with previous reports.^23,45^ In addition, no secondary peaks indicative of micelle formation could be detected, suggesting that DSPE-PEG was not ejected under these conditions. However, given the limited resolution of DLS, the presence of large micelles, bicelles, nanodiscs, or other PEG-rich nanostructures whose sizes overlap with the primary liposome population could not be ruled out. Similarly, subtle nanoscale structural alterations, such as bilayer thinning, would remain undetectable by DLS but could still contribute to cargo escape. Complementary techniques were therefore required to resolve these nanoscale features.

To assess liposome structural integrity and overcome the resolution limitations of light scattering, SANS measurements were performed before and after US insonation. SANS is highly sensitive to bilayer thickness and nanoscale features, enabling detection of subtle structural variations that are not accessible by DLS.^46^ Each formulation containing a distinct lipopolymer exhibited a characteristic scattering profile (Figure 3A–3D), and the fits yielded bilayer structures consistent with expectations (Table S1). More importantly, scattering profiles remained unchanged following US insonation across all formulations, regardless of release efficiency, with no shifts in features or deviations requiring alternative fitting (Figure 3A–3D). The fitted bilayer thickness and scattering length densities were therefore near-identical before (Table S1) and after (Table S2) insonation across all formulations, as exemplified by the thickness of the hydrocarbon chains of the lipid bilayer for the PEG formulation being 31.6 ± 0.3 Å and 30.8 ± 0.3 Å before and after insonation, respectively. This indicates that neither significant bilayer thinning nor formation of PEG-rich micellar particles occurred, as distinct scattering profiles would otherwise be observed.^47^ The retention of lipopolymers within the bilayer is thermodynamically consistent with the strong hydrophobic interactions of the long, saturated acyl chains of DSPE.^48^ Overall, while these static measurements cannot capture the transient bilayer dynamics occurring during insonation, these observations show that US triggering does not induce permanent structural changes in the bilayer, consistent with a model in which US-triggered release occurs via localized, transient, and reversible sonoporation through reorganization of PEGylated lipids.

**Figure 3:**
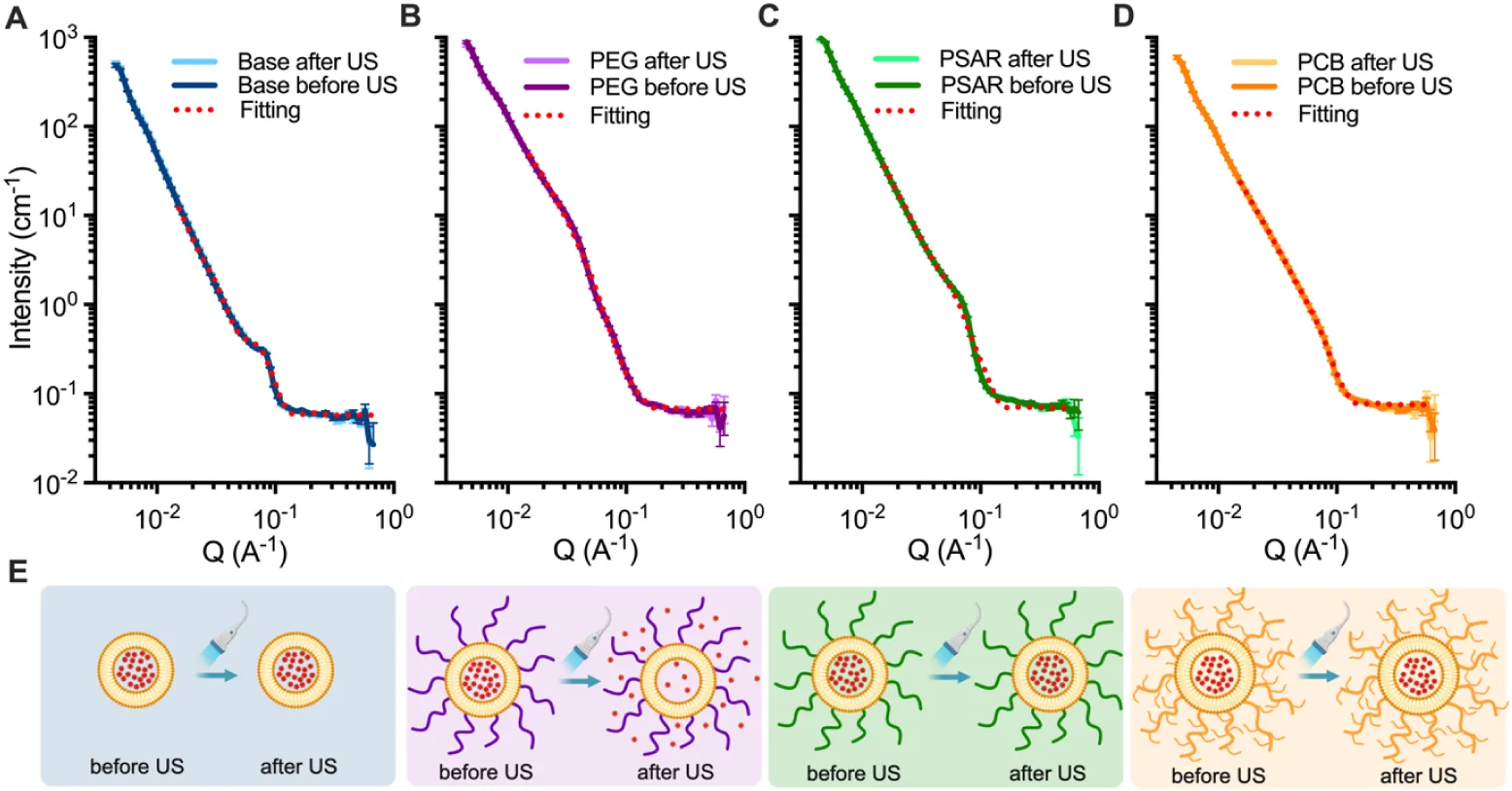
Liposome structure before and after insonation as assessed by SANS. (A-D) SANS profiles of (A) bare, (B) PEG-, (C) PSAR-, and (D) PCB-coated liposomes before (darker lines) and after (lighter lines) US exposure (3 W·cm^–2^ intensity, 1 MHz frequency, 50% duty cycle, 1 Hz pulse repetition frequency, 10 min). Details of the fitting procedures and the resulting parameters are provided in the SI Methods and Table S1, respectively. (E) Schematic representations of each formulation, illustrating preserved membrane nanostructure following insonation and highlighting enhanced release solely in PEGylated liposomes. Although not depicted for clarity, the polymer chains are also present in the inner leaflets of the bilayer as a consequence of the extrusion-based preparation method. Schematics in (E) created with Biorender.com.

Directly observing nanoscale dynamics under US insonation is experimentally challenging. To gain molecular-level insights into localized, transient events driven by PEGylated lipids that could enhance US-triggered cargo release, we employed CG-MD simulations. Using pressure perturbation protocols adapted from prior MD studies that investigated US-like pressure perturbations in lipid bilayers,^19,49^ we observed the formation of pores in which PEGylated lipids preferentially adopted micelle-like conformations at the pore rims (Figure 4). Such pores spanned approximately 3 to 10 nm in diameter. While this range should be interpreted with caution, given the differences between CG-MD pressure perturbations and a real ultrasound environment and the known dependence of pore dimensions on force field resolution,^50^ it is consistent with previous studies^24,51,52^ and sufficient to permit passage of calcein. Together with SANS evidence showing intact bilayers, these observations are consistent with a sonoporation model in which transient DSPE-PEG reorientation occurs at pore rims (Figure 4B and 4C). Facilitated by their low CMC (Figure 2G), PEGylated lipids arrange into micelle-like structures in US-induced pores that would contribute to enhanced cargo release while preserving overall membrane integrity. In contrast, lipopolymers or lipids with higher CMC values, such as PCB, PSAR, or the base formulation, would be expected to adopt such pore-associated conformations less readily, which would explain their lower US-triggered release. It should be noted that this interpretation remains inferential at this stage because analogous CG-MD simulations for PSAR and PCB were not performed, given the absence of validated Martini3 force-field parameters for these lipopolymers (the development of which falls outside the scope of the present work). In addition, the onset pressures in the CG-MD simulations were higher than those applied experimentally, owing to differences in how mechanical stress is imposed in simulations compared with an actual ultrasound transducer. The simulations therefore offer a qualitative view of pressure-induced bilayer response rather than a quantitative assessment. Consistent with prior studies of membrane tension-driven pore formation,^50,51,53^ they support transient pore formation as the dominant PEG-facilitated mechanism in ultrasound-triggered release.

**Figure 4:**
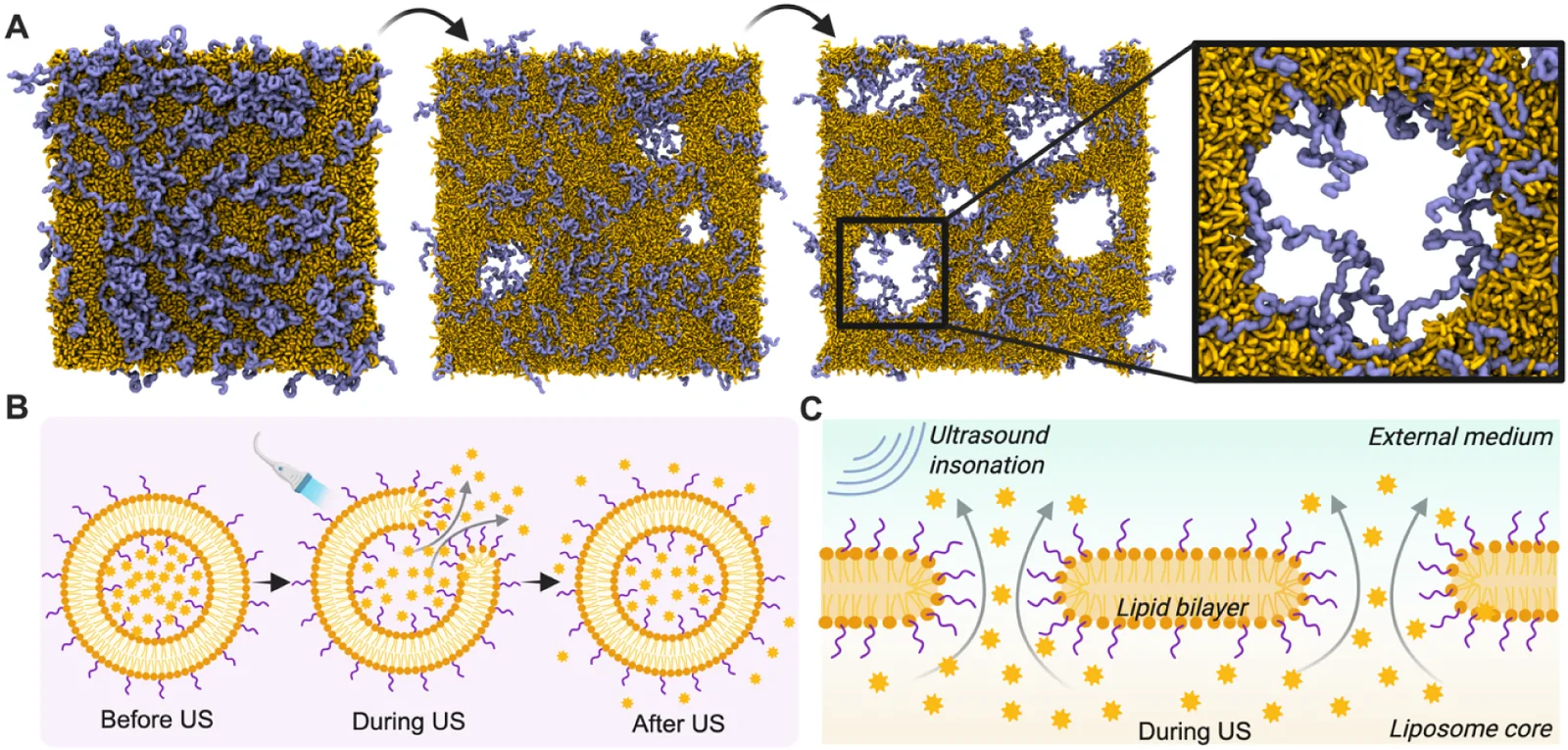
Lipid bilayer response to pressure perturbation. (A) CG-MD snapshots showing transient sonoporation in the lipid bilayer. Snapshots correspond to *t* = 0, 15, and 24 ns. Water beads are omitted for clarity, images are not shown at scale. Yellow represents the hydrophobic membrane components (DOPC, DSPC, DSPE, and cholesterol), while purple denotes the PEG chains from the PEGylated lipids. The zoom-in highlights the PEG arrangement in the pressure-induced pores. (B) Schematic of a PEGylated liposome before, during, and after US insonation, illustrating release through PEG-driven transient openings while preserving bilayer integrity. (C) Side-view schematic of a lipid bilayer under US, showing PEG-mediated sonoporation enabling release of cargo in the liposomal aqueous core into the external medium. Schematics (B) and (C) created with Biorender.com.

### Implications for the Design of More Effective US-Responsive Liposomes beyond PEG

Collectively, our results indicate that the PEG-driven mechanism underlying enhanced US-triggered release from liposomes cannot be readily explained by changes in bulk bilayer properties such as differences in PP, membrane mechanics, thermal behavior, or fluidity. Similarly, structural changes, including micelle formation due to PEG lipopolymer ejection or bilayer thinning, do not appear to occur upon insonation. Instead, experiments and simulations suggest that DSPE-PEG adopts micelle-like conformations at US-induced pore rims, facilitated by its low CMC, which could promote transient sonoporation in the lipid bilayer and facilitate cargo release. These insights suggest a shift in the design of US-responsive liposomes away from empirical optimization and bulk bilayer considerations toward controlling how lipid and lipopolymer composition modulate poration under US perturbation (Figure 5).^54,55^

**Figure 5:**
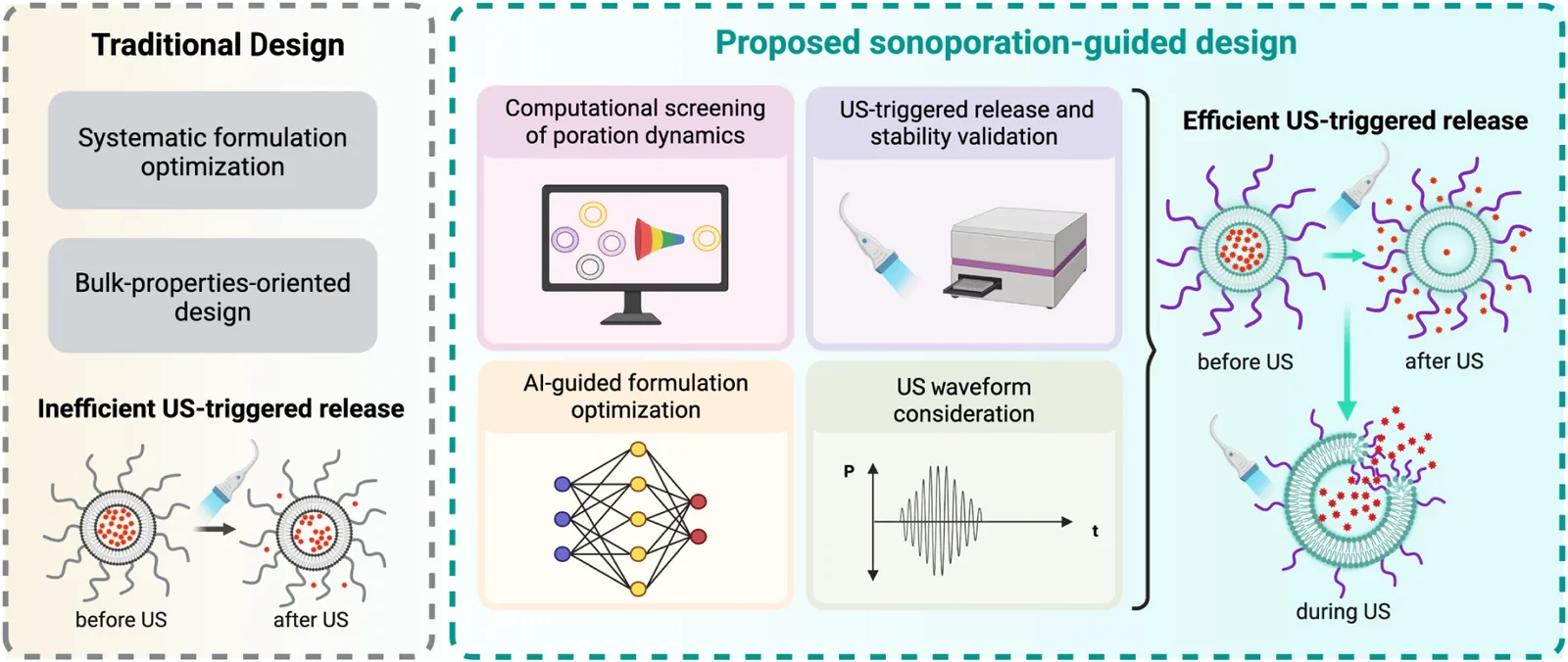
Sonoporation-guided design strategy for US-responsive liposomes with enhanced release efficiency. Traditional liposome design, based on bulk bilayer properties and empirical optimization, often yields inefficient US-triggered release. The proposed sonoporation framework, to enable the design of liposomes with more efficient US-triggered release, emphasizes (i) computational screening of pore-formation energetics for first-pass formulation screening, (ii) experimental evaluation of ultrasound-triggered release efficiency and stability under physiologically relevant conditions, (iii) AI-guided identification of nonobvious formulations that enhance US-triggered release efficiency, and (iv) acoustic waveform considerations to align acoustic parameters with formulation optimization. Schematic created with Biorender.com.

A sonoporation-oriented framework could provide a basis for linking the PEG-focused insights of this work to broader trends in composition-dependent, US-triggered release reported in the literature. For example, lipids with phosphatidylcholine (PC) and phosphatidylglycerol (PG) headgroups have been reported to induce higher US-triggered release than phosphatidylethanolamine (PE) or phosphatidic acid (PA).^16^ While this was initially attributed to variations in thermal stability and *T*_m_ shifts, our results suggest that such an interpretation may be incomplete. Instead, considering how lipid and lipopolymer composition facilitates pore formation upon insonation could offer a more plausible explanation. This interpretation aligns with recent atomistic MD simulations in which PC and PG headgroups exhibited lower pore nucleation energies and reduced line tension than PE or PA^54^, consistent with the higher US-triggered release efficiencies observed experimentally.^16^ Similarly, this rationale could align with the design of the patented US-responsive formulation IMP-301, in which the authors showed that lysolipids accumulate at pore rims upon insonation, thereby facilitating cargo release.^19,56^ This fits within the sonoporation-oriented framework, as recent computational studies have shown that lysolipids lower pore nucleation energies and reduce line tension.^54^ More broadly, other low-CMC lipopolymers or surfactants might enable enhanced US-triggered release, as demonstrated for liposomes containing oligo­(ethylene glycol) surfactants.^22^ However, this influence is unlikely to be monotonic, as a too-high fraction of low-CMC lipopolymer (e.g., ∼20 mol % DSPE-PEG) would likely yield a heterogeneous mixture of morphologies such as micelles, bicelles, and liposomes,^57^ instead of the desired uniform liposome population. Taken together, the PEG-related insights presented here suggest that lipid and lipopolymer composition should be evaluated in terms of their impact on transient sonoporation—rather than solely on bulk bilayer properties—to better rationalize and guide the design of more effective US-responsive liposomes.

Building on this perspective, computational evaluation of the energetics of pressure-induced pore formation could provide an efficient first-pass screening strategy to identify promising US-responsive formulations (Figure 5). By focusing on sonoporation and the energetic landscape of pore formation—rather than optimization based on bulk bilayer properties—such an approach could accelerate the assessment of liposome compositions for enhanced US-triggered release. Specifically, potential-of-mean-force calculations, recently demonstrated to extract key energetic parameters such as the nucleation free energy and pore line tension,^54^ could be employed to evaluate the poration susceptibility of different liposomal formulations, directly informing the selection of candidates with enhanced US-triggered release within the proposed sonoporation framework. At the same time, achieving a balance between favorable ultrasound-induced pore-formation thermodynamics and desired stability in the absence of ultrasound would be essential, with experimental validation serving as the ultimate benchmark. For example, high molar ratios of short-chain or unsaturated lipids may facilitate poration^54^ and thereby enhance US-triggered release, but could also compromise structural integrity and cargo retention stability. Thus, computational screening would require integration with experimental evaluation of membrane stability, cargo retention, and ultrasound-triggered release in complex fluids (e.g., serum) under physiologically relevant conditions to guide the development of more effective US-responsive liposomes (Figure 5).

Although computational analysis of US-induced pore formation energetics may streamline early-stage screening of liposomal formulations, multicomponent systems containing diverse phospholipids, sterols, and lipopolymers could exhibit nonlinear, coupled interactions, creating a high-dimensional design space that could become computationally intractable. In this setting, data-driven and AI-enabled approaches could identify liposomal compositions with enhanced US-triggered release (Figure 5).^58^ For example, lipids could be encoded by physicochemical descriptors (e.g., headgroup chemistry, chain length, and degree of unsaturation), as previously done for ionizable lipids in lipid nanoparticles,^59^ or by whole-molecule descriptors. Combined with in-house or literature-reported release data, these could train a predictive model to propose formulations for subsequent experimental validation. However, the effectiveness of such models would depend on access to large, standardized data sets.^60^ While sample-efficient strategies such as Bayesian optimization may remain feasible in low-data regimes, more data-intensive models such as deep neural networks would require large, well-curated training sets, which are only accessible. This is done through high-throughput experimentation,^61^ self-driving robotic automation,^62^ or collaborative databases.

Beyond liposome formulation, acoustic parameter selection is an equally important determinant of US-triggered release. While the release profiles reported here were obtained under a single set of US parameters, our recent study demonstrated that acoustic parameters, including frequency, pressure, duty cycle, and pulse repetition frequency, strongly modulate US-triggered release.^63^ Such a study, however, used a single liposomal formulation. Mechanistically, formulation and acoustic parameters would be expected to interact in a coupled manner such that different US conditions could either attenuate or amplify the formulation-dependent differences in release observed in this work. This coupling arises because pore formation and closure dynamics are sensitive to both bilayer composition, through modulation of the pressure threshold required to initiate pore formation (*P*_THR_), and the applied acoustic field, through the temporal and spatial distribution of US waves above such *P*_THR_, as discussed in our recent work.^63^ For example, a structured design of experiments across acoustic parameters (e.g., frequency, pressure amplitude, duty cycle, and pulse repetition frequency) and a representative set of liposomal formulations would be a natural and actionable next step, mapping the coupled influence of acoustic and compositional variables on US-triggered release.

Finally, while the use of PBS and calcein-loaded liposomes provides a simplified system for the mechanistic focus of this work, the transition to *in vivo* environments introduces additional factors that could modulate release. Upon systemic administration, liposomes rapidly acquire a protein corona,^64^ which can modulate liposome-US interactions in a composition-dependent manner^65^ and may differentially affect formulations based on their surface chemistry, in addition to the formulation-dependent accelerated blood clearance phenomenon. Similarly, while calcein served here as a convenient hydrophilic model cargo, alternative therapeutic payloads may exhibit distinct, formulation-dependent release kinetics under insonation,^45^ requiring independent validation for each cargo-formulation combination. Overall, effective clinical translation of US-responsive liposomes will require jointly optimizing liposome formulation and acoustic parameters (Figure 5) within the constraints imposed by *in vivo* environments, a challenge for which the mechanistic and sonoporation-oriented framework proposed here aims to provide a conceptual foundation.

## Conclusions

In summary, this work investigates the ability of DSPE-PEG to enhance US-triggered release from liposomes. By comparing release profiles and systematically characterizing PEGylated formulations compared with liposomes incorporating alternative stealth polymers such as PCB or PSAR, we show that the PEG-driven increase in US-triggered release cannot be attributed to previously hypothesized differences in bulk bilayer properties, including PP, membrane mechanics, thermal behavior, or fluidity. Similarly, structural variations, such as DSPE-PEG ejection as micelles or bilayer thinning, did not occur upon insonation. Instead, our results suggest that PEG-containing lipids, due to their markedly low CMC, promote sonoporation by forming micelle-like structures at pore rims under acoustic perturbation, ultimately leading to a higher US-triggered release. Finally, we discuss how these insights could be extended beyond PEG-lipopolymers to guide the rational design of more efficient US-responsive liposomes by redirecting optimization efforts from bulk bilayer property optimization toward a sonoporation-oriented rationale, integrating computational screening, AI-enabled design, and acoustic waveform considerations.

## Materials and Methods

### Liposome Synthesis and Physicochemical Characterization

Liposomes were prepared by the thin-film hydration method.^66^ DSPC, DOPC, cholesterol, and DSPE-PEG_MW=2000_ were purchased from Avanti Polar Lipids. DSPE-PCB_MW=5700_ and DSPE-PSAR_MW=2200_ were obtained from Ruixi Biotech. Briefly, DSPC, DOPC, cholesterol, and DSPE-lipopolymer (48:22:25:5 mol %) were dissolved in 2:1 chloroform:methanol at a total lipid concentration of 15 mg/mL and transferred to a round-bottom flask. The solvent was removed by rotary evaporation (Rotavapor R-100, Buchi) at 40 °C under stepwise reduced pressure (600 mbar, decreased by 100 mbar every 120 s to 15 mbar), then dried under high vacuum (CRVpro 4, Welch) for 3 h to remove residual solvent. The resulting lipid film was hydrated with 2 mL of 50 mM calcein solution, which was prepared in PBS (10 mM, pH 7.4) at 60 °C for 20 min under continuous agitation. The dispersion was subjected to four freeze–thaw cycles (1 min in liquid nitrogen and 4 min at 60 °C) and subsequently extruded (Mini-Extruder, Avanti Polar Lipids) 15 times through 100 nm polycarbonate membranes in a heating block at 67 °C. Nonencapsulated calcein was removed by dialysis (MWCO 1000 kDa) against PBS (10 mM, pH 7.4, at 4 °C), with repeated buffer exchange until the external solution was free of calcein.

Lipid concentration was quantified using the Stewart assay.^67^ Briefly, 10 μL of liposome dispersion was mixed with 2 mL of chloroform, vortexed for 1 min, mixed with 2 mL of ferrothiocyanate reagent, vortexed for 1 min, and centrifuged (2000 rpm, 10 min). The organic phase was recorded using UV–vis between 400 and 550 nm (DR5000, Hach Lange), and the concentration was obtained against a calibration curve of lipid standards. Hydrodynamic diameter and PDI were determined by DLS (Zetasizer Nano ZS, Malvern Panalytical) at 25 °C, following 120 s equilibration using 173° backscatter detection (three runs per sample; 0.1 mg lipid/mL of 10 mM PBS). ζ-potential measurements were performed in 1 mM PBS (0.1 mg lipid/mL) using capillary cells (DTS1070, Malvern Panalytical).

### Ultrasound-Triggered Release and Stability Studies

A 2 mL aliquot of liposome dispersion at 0.1 mg/mL lipid content in PBS (10 mM, pH 7.4), was placed in a 5 mL glass vial sealed with Parafilm M. US gel (Aquasonic 100) was deposited on the transducer for acoustic coupling, and the vial was placed on the gel. US was delivered using a New Pocket device (New Age) with a transducer operating at a frequency of 1 MHz, an intensity of 3 W·cm^–2^, a 50% duty cycle, and a 1 Hz pulse repetition frequency. After US treatment, each sample was transferred to a disposable cuvette (10 mm, Fisherbrand) and analyzed fluorometrically using a J-815 CD spectrometer (JASCO). Excitation was set at 490 nm, and emission was collected from 500 to 600 nm with a 5 nm bandwidth and a 4 ms detector response time. The release percentage was determined using R(%) = [(*F* – *F*_n_)/(*F*_p_ – *F*_n_)] × 100, where *F* denotes the fluorescence intensity following insonation, *F*_n_ represents the untreated negative control, and *F*_p_ corresponds to the fully disrupted positive control prepared by adding 10 μL of Triton X-100 to 2 mL of liposome suspension and vortexing for 1 min. Experiments were performed across independently prepared lipid batches, with each time point corresponding to an independent ultrasound run; minor variations in lipid preparation and ultrasound exposure may contribute to experimental variability, especially for samples with higher release at longer insonation times (Figure 1A). Liposome size and PDI were measured before and after insonation. The temperature during US exposure was monitored at the center of the cuvette using a thermocouple connected to a 179 True-RMS multimeter (Fluke).

Thermal incubation was performed for a liposome dispersion at 0.1 mg/mL lipid content in PBS (10 mM, pH 7.4), at 37 °C in a temperature-controlled water bath (ETS-D5 with RET Basic C, IKA). Samples were equilibrated at room temperature for 20 min before fluorescence measurements in 10 mm disposable cuvettes (Fisherbrand). Storage stability was monitored at 4 °C by measuring hydrodynamic diameter and PDI using DLS, as previously mentioned, and cargo retention using fluorescence spectroscopy (JASCO), with samples equilibrated to room temperature for 10 min before measurement.

### AFM Nanoindentation

Glass coverslips were sequentially rinsed with deionized water, ethanol, and deionized water, then dried under a nitrogen stream. A DSPE-PEG solution (5 mg/mL in chloroform) was deposited (100 μL per substrate) onto substrates maintained at ∼40 °C, and solvent evaporation yielded uniform monolayers. Liposome suspensions at 0.05 mg/mL were deposited onto the coated substrates and allowed to adsorb before imaging and mechanical characterization in liquid (PBS, 10 mM). AFM experiments were conducted in liquid using a Bruker Dimension Icon instrument operated in PeakForce QNM mode. MLCT-Bio Triangular C cantilevers (Bruker) with nominal spring constants of 0.01 N/m and a tip radius of 20 nm were used. Cantilever deflection sensitivity was determined from force ramps on a sapphire standard, and exact spring constants were obtained by thermal tuning before measurements. Imaging was conducted at a PeakForce frequency of 1 kHz using low interaction forces (≤1 nN) to identify intact liposomes before indentation. Force–distance curves were then acquired at the center of selected liposomes at a ramp rate of 1 Hz with a maximum applied force of 300 pN to stay in the elastic regime. Force curves were baseline-corrected so that the zero-force level corresponded to no interaction between the tip and the sample and converted to force–indentation data for analysis. The curves were fitted with the Hertz model^68^ with a Poisson’s ratio of 0.5, and the stiffness was determined from the slope of the force–indentation curve. Image processing and force-curve analyses were performed using NanoScope Analysis (v3) and Gwyddion (v2.67).

### Differential Scanning Calorimetry

DSC measurements were performed using a PerkinElmer Pyris Calorimeter purged with high-purity nitrogen at a constant flow rate of 20 mL/min. Temperature and heat-flow calibrations were conducted prior to measurements using indium as a standard, and scans were performed at a heating rate of 2 °C/min. Approximately 30 μL of liposome suspension at 20 mg/mL lipid content was transferred into 40 μL aluminum crucibles (PerkinElmer), weighed using a microbalance, and hermetically sealed with matching aluminum lids. Because the multicomponent formulations (DSPC:DOPC:DSPE-polymer with cholesterol) would not be expected to have a well-defined *T*_m_, measurements were conducted using simplified binary DPPC/DSPE-polymer formulations to evaluate the effect of DSPE-polymer in shifting *T*_m_. An identically sealed empty pan served as the reference. The sample and reference pans were placed in their respective holders in the DSC furnace, and the thermal program consisted of an initial isothermal equilibration at 25 °C for 5 min, followed by heating from 20 to 60 °C at 2 °C/min, and an isothermal hold at 60 °C for 5 min. Heat-flow data were normalized to sample mass before analysis.

### Fluorescence Polarization

Fluorescence polarization measurements were performed using DPH as a hydrophobic probe following established protocols.^38^ Briefly, DPH was dissolved in tetrahydrofuran to obtain a 1 mM stock solution. Liposomes were diluted to 0.05 mg/mL in PBS (10 mM, pH 7.4). The DPH stock was added to the liposome suspension at a fixed lipid:DPH volume ratio of 500:1 (v/v). Samples were gently mixed, incubated for 90 min at room temperature, and then equilibrated overnight in the dark. Fluorescence polarization measurements were carried out in 96-well plates using a Synergy H1 microplate reader (BioTek Instruments). Excitation was performed at 360 nm with vertically polarized light, and emission was collected at 460 nm in both the parallel (I_∥_) and perpendicular (I_⊥_) orientations relative to the excitation plane. Measurements were conducted from 25 to 45 °C in 2 °C increments, with a 30 min equilibration period at each temperature before acquisition; samples were pre-equilibrated at 25 °C before the initial reading. Polarization was calculated according to P= (I_∥_-G·I_⊥_)/(I_∥_+G·I_⊥_), where the G-factor provided by the manufacturer was experimentally verified and found to be 1.

### Small-Angle Neutron Scattering

SANS measurements were performed at room temperature on the ISIS Neutron and Muon Source (STFC Rutherford Appleton Laboratory, Didcot, UK) using the Larmor time-of-flight instrument. Liposome dispersions (12.5 mg/mL total lipid) were prepared in 100% D_2_O and loaded into 1 mm path-length quartz cuvettes (350 μL; Type 110-QS, Hellma Analytics) sealed with PTFE stoppers. Measurements were acquired before and immediately after 10 min of US insonation, performed under conditions identical to the US-triggered release experiments described above. An incident neutron wavelength band of 0.9–13 Å provided a simultaneous scattering vector (*Q*) range of 0.005–0.7 Å^–1^ where *Q* = (4π/λ)·sin­(θ), λ is the neutron wavelength, and 2θ is the scattering angle. Two-dimensional scattering patterns were reduced using Mantid^69^ and corrected for detector efficiency, sample transmission, and background scattering from the quartz cell and the solvent. Model fitting was performed in SasView (v6.1.0)^70^ and is described in the SI Methods.

### Critical Micelle Concentration Determination

CMC was determined using a fluorescence probe method adapted from previous reports.^71,72^ Briefly, DSPE-polymer conjugates were dissolved in a 3:1 chloroform:methanol mixture and diluted to the desired concentrations. Organic solvents were evaporated under ambient conditions overnight to form uniform, dry lipid films. Films were rehydrated with 2 mL of 10 mM PBS and incubated with 5 μL of 1 mM DPH in ethanol (final probe concentration: 2.5 μM). Samples were vortexed vigorously for 3 min, sonicated in a bath sonicator for 5 min, and equilibrated at room temperature for 30 min before analysis. Fluorescence emission spectra were collected from 390 to 600 nm at an excitation wavelength of 360 nm, with a 4 nm bandwidth and a response time of 4 ms, using a J-815 CD spectrometer (JASCO). The CMC was quantified as the inflection point of the fluorescence intensity against concentration curve.

### Coarse-Grained Molecular Dynamics

A coarse-grained lipid bilayer composed of DSPC:DOPC:cholesterol:DSPE-PEG2000 (48:22:25:5 mol %), matching the experimentally synthesized composition, was generated using INSANE^73^ and Polyply^74^ within the Martini 3 force field framework.^75^ The membrane was placed in a simulation box of 40 × 40 × 20 nm^3^ and solvated with 70,381 Martini water beads (water-to-lipid ratio ≈ 13:1). The anionic DSPE-PEG2000 components were charge-balanced with 270 Na^+^ counterions. All molecular dynamics simulations were carried out using GROMACS (version 2025.1).^76,77^ Energy minimization was performed using the steepest descent algorithm. Equilibration was conducted in a stepwise manner employing the leapfrog integrator, adapted from a previous protocol.^78^ Electrostatic interactions were treated using the reaction-field approach (cutoff of 1.1 nm, relative dielectric constant ε_r_ = 2.5), and van der Waals interactions were described using a potential-shifted Verlet scheme with a 1.1 nm cutoff.^79,80^ Initial equilibration consisted of three consecutive NVT runs using the v-rescale thermostat (τ_t_ = 1 ps),^81^ during which the time step was gradually increased from 1 fs (5 ps total simulation time) to 5 fs (100 ps) and subsequently to 10 fs (400 ps). This was followed by a 4 ns NPT equilibration using semi-isotropic c-rescale pressure coupling (τ_p_ = 10 ps) with a 20 fs time step.^82^ Pressure-perturbation production simulations were performed, adapted from a previous report,^19^ in the NPT ensemble at 310 K using a 20 fs time step and semi-isotropic pressure coupling. The lateral pressure was maintained at 1 bar, while the pressure normal to the bilayer was set to 125 bar. Structural snapshots were visualized using MartiniGlass^83^ and Visual Molecular Dynamics,^84^ with water and ion beads omitted for clarity.

### Statistics

Statistics were performed with GraphPad Prism v11 using ordinary one-way ANOVA or unpaired Welch’s *t*-test unless otherwise stated. All data in plots are shown as means and standard deviations unless otherwise stated.

## Supplementary Material



## Data Availability

All data are available in the main text or the Supporting Information. Additional data related to this paper may be requested from the authors.

## Notes

SANS fitting methodology using SasView (Supporting Information, Methods); physicochemical characterization of liposomes (Figure S1); effect of lipopolymer molar ratio on ultrasound-triggered release and temperature increase during insonation (Figure S2); liposome size and PDI before and after insonation (Figure S3); schematic representations of the SANS fitting models for bare, PEG-coated, PSAR-coated, and PCB-coated liposomes (Figure S4); and SANS fitting parameters for bare, PEG-coated, PSAR-coated, and PCB-coated liposomes before (Table S1) and after (Table S2) insonation (PDF)

## Abbreviations

US: ultrasound
PEG: polyethylene glycol
PSAR: polysarcosine
PCB: poly­(carboxybetaine)
DSPE: 1,2-distearoyl-*sn*-glycero-3-phosphoethanolamine
PP: packing parameter
*T*_m_: transition temperature
AFM: atomic force microscopy
DSC: differential scanning calorimetry
CMC: critical micelle concentration
PDI: polydispersity index
SANS: small-angle neutron scattering
CG-MD: coarse-grained molecular dynamics
AI: artificial intelligence
DSPC: 1,2-distearoyl-*sn*-glycero-3-phosphocholine
DOPC: 1,2-dioleoyl-*sn*-glycero-3-phosphocholine
DPH: 1,6-diphenyl-1,3,5-hexatriene
DLS: dynamic light scattering
PC: phosphatidylcholine
PG: phosphatidylglycerol
PE: phosphatidylethanolamine
PA: phosphatidic acid
