# Supplementary material for: Unraveling the Mechanism of PEG-Enhanced Ultrasound-Triggered Release from Liposomes
Source: Langmuir. 2026 Sep 3;42(36):26595–606. doi: 10.1021/acs.langmuir.6c03550 (PMC13585128; doi:10.1021/acs.langmuir.6c03550)
Supplement: Supplementary file 1 [file la6c03550_si_001.pdf]

Supporting Information

Unraveling the Mechanism of PEG-Enhanced Ultrasound-Triggered Release from Liposomes

Ignasi Simon[1,2], Farzaneh Gholamianpour[1], Lassi V. Tiihonen[3], Simon Vliek[1], Amber Renkema[1], Teun L.A. Janssen[3], Coen Fransen[3], Steven R. Parnell[4], Riccardo Alessandri[5], Remco Hartkamp[6], Wim G. Bouwman[3], Tiago L. Costa[2], and Alina Y. Rwei[1,]*

[1]Department of Chemical Engineering, Delft University of Technology, 2629 HZ Delft, the Netherlands, [2]Department of Microelectronics, Delft University of Technology, 2628 CD Delft, the Netherlands, [3]Department for Radiation Science and Technology, Delft University of Technology, 2629 JB Delft, the Netherlands, [4]ISIS Neutron and Muon Source, Rutherford Appleton Laboratory, Oxon OX11 0QX Chilton, United Kingdom,[5]Department of Chemical Engineering, KU Leuven, 3001 Leuven, Belgium, [6]Department of Process & Energy, Delft University of Technology, 2628 CB Delft, the Netherlands.

[*]To whom correspondence may be addressed. A.Y.Rwei@tudelft.nl (Alina Y. Rwei),

SUPPORTING INFORMATION METHODS

Small-Angle Neutron Scattering (SANS) fitting with SasView

SANS data were analyzed using SasView (v6.1.0) using the spherical core multi-shell model. Polymer-functionalized liposomes (i.e., poly(ethylene glycol) (PEG)-, poly(sarcosine) (PSAR)-, and poly(carboxybetaine) (PCB)-coated) were described using a five-shell structure consisting, from inside to outside, of a polymeric layer, headgroup layer, hydrophobic lipid bilayer core, headgroup layer, and outer polymeric layer (Figure S4A). The base formulation was initially modeled using a three-shell structure (headgroup layer/hydrophobic bilayer core/headgroup layer) as the preparation protocol was expected to yield predominantly unilamellar vesicles. However, this model did not fully reproduce the experimental data due to the apparent presence of a multilamellar peak, consistent with previous reports showing some multilamellarity for non-stealth formulations, likely due to the absence of bulky polymers that hinder lamellar stacking, which disappeared upon stealthing.[1] A four-lamellar model instead provided a significantly improved fit (Figure S4B). For layers containing mixed lipids and cholesterol, scattering length densities (SLDs) were calculated as molar-weighted averages of the constituent components of the experimental formulation. Components neutron SLDs were obtained either from molecular composition – using coherent neutron scattering lengths and molecular volumes – or from literature values[2,3] and were as follows: D_2O ($6.384 \cdot 10^{-6}$ Å^{-2}), phosphatidylethanolamine headgroup ($1.877 \cdot 10^{-6}$ Å^{-2}), phosphatidylcholine headgroup ($1.877 \cdot 10^{-6}$ Å^{-2}), oleoyl (18:1c) and palmitoyl (16:0) acyl chains ($-0.386 \cdot 10^{-6}$ Å^{-2}), and cholesterol ($0.386 \cdot 10^{-6}$ Å^{-2}). Hydration of the headgroup was considered by defining an effective SLD (SLD_{eff}) that included a volumetric hydration fraction (φ), $SLD_{eff} = SLD_{material} \cdot (1 - \varphi) + SLD_{D2O} \cdot \varphi$, with $\varphi = 0.3$ for headgroup layers, while the hydrophobic core was assumed non-hydrated. The SLD of the polymeric layers was freely fitted. All fits were performed over a Q-range of 0.015-0.67 Å$^{-1,}$ and the resolution of the Larmor instrument was accounted for using the provided dQ-values. To reduce parameter correlation and ensure physically meaningful fits, several parameters were fixed based on prior knowledge of the system (Table S1). In particular, SLDs of lipid-based layers and solvents were

calculated directly from the molecular composition of the formulations, as described above. Layer thicknesses associated with lipid headgroup and bilayer hydrophobic core were fixed based on typical phospholipid dimensions. For the multilamellar fit of the base formulation, thickness distributions for solvent-associated layers were approximated by fixing the nominal thickness to 20 Å to represent the minimal repeat unit of the lamellar structure rather than a vesicle radius, with structural variation instead captured by fitting polydispersity (PD) values > 1 using a Gaussian distribution, yielding effective one-sided Gaussian distributions whose mean thickness is defined by the PD parameter. For the unilamellar fits of the polymer-functionalized liposomes, the core radii were fitted with a lognormal polydispersity distribution with PD << 1. Weighted least-squares fitting was performed in SasView using the experimental intensity uncertainties (ΔI), from which the reduced chi-square (χ^2) was obtained. Sample-specific parameters and corresponding χ^2 are provided in Table S1.

SUPPORTING INFORMATION FIGURES AND TABLES

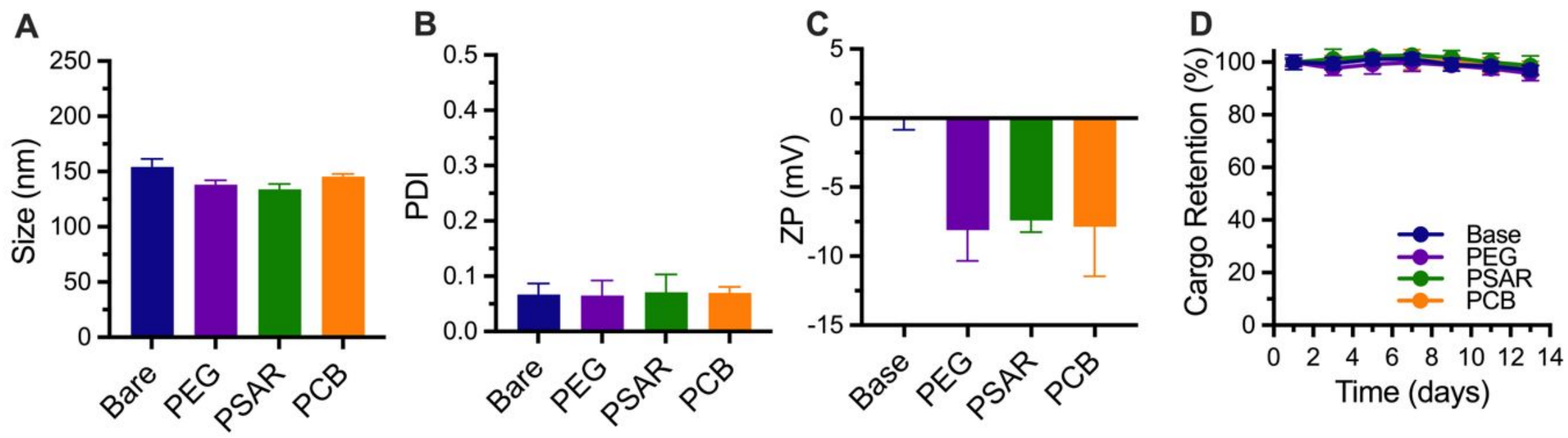

Figure S1. Physicochemical characterization of bare and polymer-coated liposomes. (A) Hydrodynamic size measured by dynamic light scattering (DLS), (B) polydispersity index (PDI) values, and (C) zeta potential for bare, PEG-, PSAR-, and PCB-coated liposomes. (n=3) (D) cargo retention under storage conditions at 4 °C for bare, PEG-, PSAR-, and PCB-coated liposomes. (n=3)

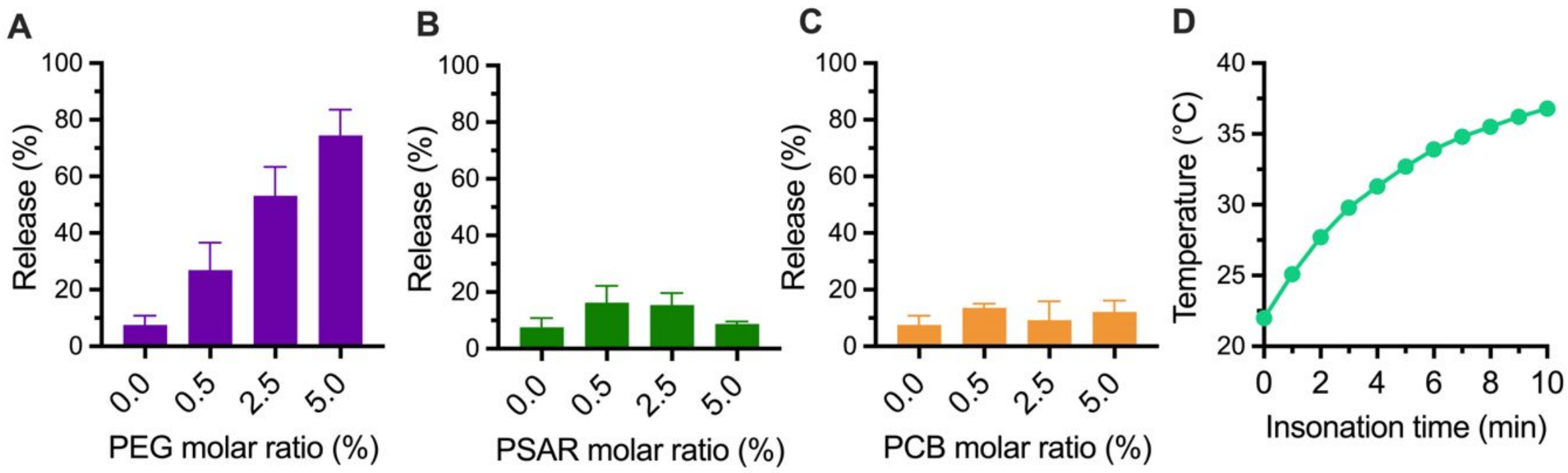

Figure S2. Influence of lipopolymer composition on US-triggered release from liposomes, and temperature measurements during insonation. (A-C) US-triggered cargo release from liposomes as a function of (A) PEG, (B) PSAR, and (C) PCB molar ratio at 10 min insonation (3 W·cm^{-2}, 1 MHz, 50% duty cycle, and 1 Hz pulse repetition frequency) (n≥2). (D) Sample temperature increase during US exposure (3 W·cm^{-2}, 1 MHz, 50% duty cycle, and 1 Hz pulse repetition frequency), measured with a thermocouple inside the liposome-containing cuvette.

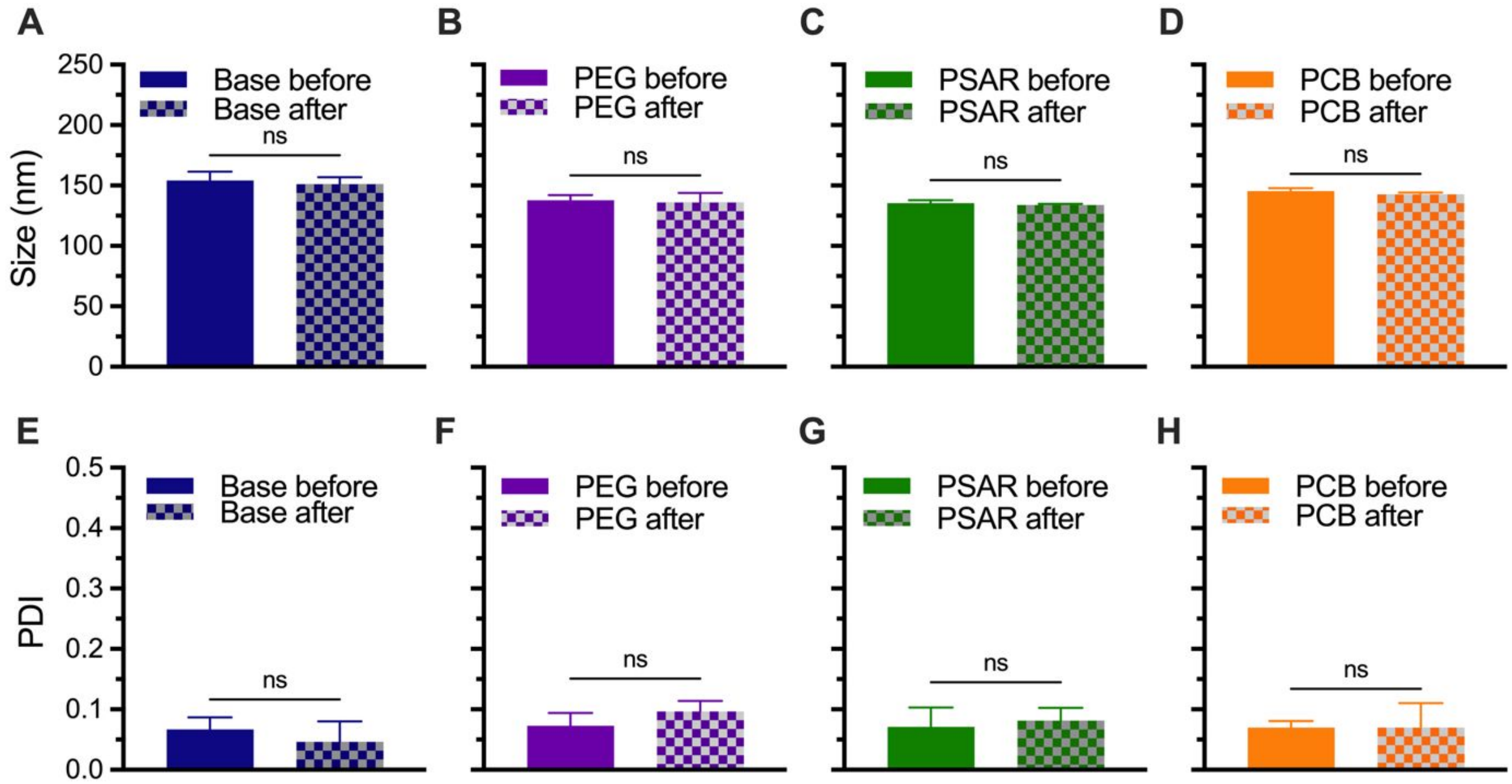

Figure S3. Liposome size and PDI before and after 10 min of US insonation (3 W·cm^{-2}, 1 MHz, 50% duty cycle, and 1 Hz pulse repetition frequency). (A-D) Hydrodynamic size measured by dynamic light scattering before and after US exposure for (A) base, (B) PEG-coated, (C) PSAR-coated, and (D) PCB-coated liposomes (n=3), showing that liposome size remains constant following insonation across all formulations, regardless of their release profile. (E-H) PDI measured by dynamic light scattering (DLS) before and after US exposure for (E) BASE, (F) PEG-coated, (G) PSAR-coated, and (H) PCB-coated liposomes (n=3), showing that PDI distribution remains equal following insonation across all formulations, regardless of their release profile.

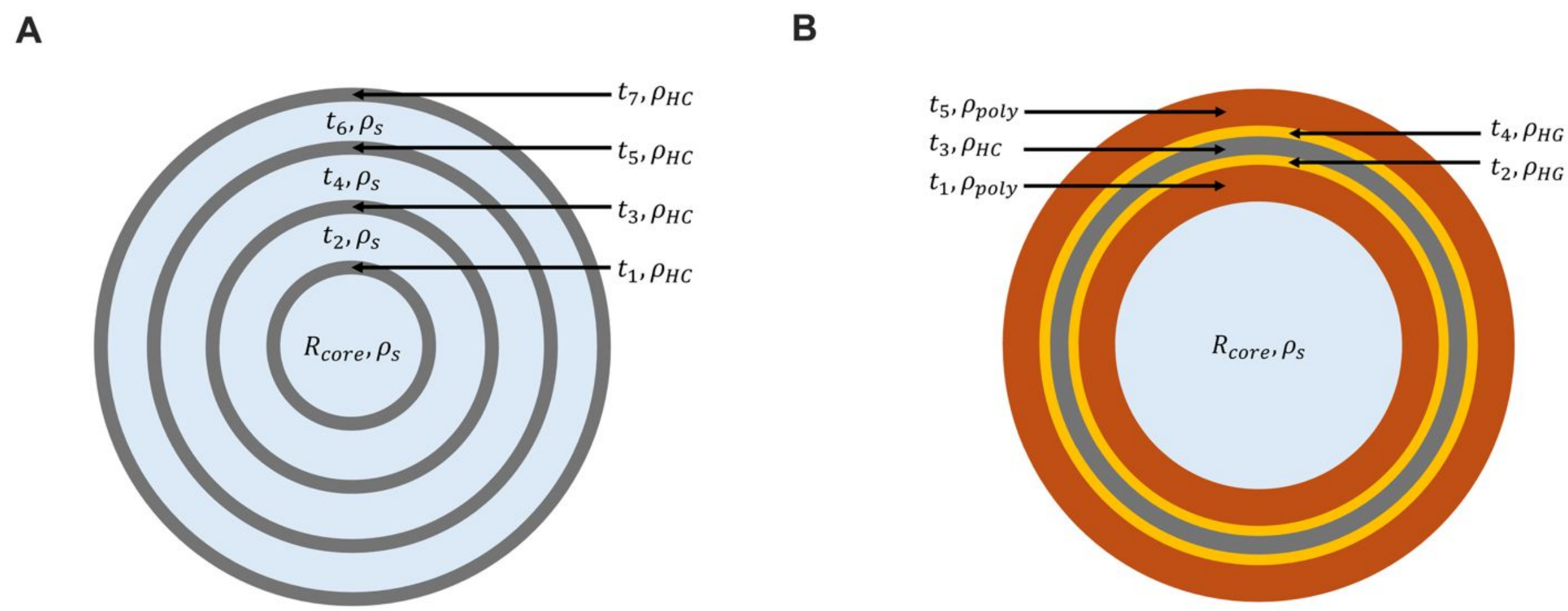

Figure S4. Schematic representations of the models used for SANS data fitting. (A) Model used to fit the base formulation, consisting of a multilamellar bilayer structure with alternating lipid bilayers and solvent layers, where each bilayer is approximated as a homogeneous slab. (B) Model used to fit the PEG-, PSAR-, and PCB-coated formulations, representing a unilamellar bilayer structure with distinct hydrocarbon chain regions, inner and outer headgroup layers, and inner and outer polymer layers. Schematics are not shown at scale, and corresponding fitted values for each formulation are reported in Table S1.

Table S1. SASView fitting parameters for bare liposomes (χ^2 = 73.2), PEG-coated liposomes (χ^2 = 380), PSAR-coated liposomes (χ^2 = 386), and PCB-coated liposomes (χ^2 = 15.2) before US insonation. *Parameters fixed during fitting. ^{a,b}Parameters constrained to equal values during fitting. Errors are reported in parentheses only for fitted parameters.

Parameter	Base	Parameter	PEG	PSAR	PCB
Scale	0.0064<br>(0.00002)	Scale	0.0549<br>(0.0004)	0.0686<br>(0.0005)	0.0388<br>(0.0006)
Background<br>[cm^{-1}]	0.0575<br>(0.0003)	Background<br>[cm^{-1}]	0.067<br>(0.005)	0.0693<br>(0.0005)	0.0754<br>(0.0004)
Core & Solvent SLD*<br>[10^{-6} Å^{-2}]	6.398	Core & Solvent SLD*<br>[10^{-6} Å^{-2}]	6.398	6.398	6.398
Core Radius*<br>[Å]	20.0	Core Radius<br>[Å]	553<br>(2)	441<br>(8)	430<br>(12)
Core Radius PD	12.2<br>(0.1)	Core Radius PD	0.070<br>(0.003)	0.18<br>(0.01)	0.199<br>(0.01)
SLD 1st Layer*<br>[10^{-6} Å^{-2}]	-0.193	SLD 1st and 5th Layer^a<br>[10^{-6} Å^{-2}]	5.42<br>(0.01)	6.175<br>(0.002)	6.293<br>(0.002)
Thickness 1st Layer*<br>[Å]	42	Thickness 1st and 5th Layer^b<br>[Å]	27.2<br>(0.2)	101.1<br>(0.3)	86.2<br>(0.9)
SLD 2nd Layer*<br>[10^{-6} Å^{-2}]	6.398	SLD 2nd and 4th Layer*<br>[10^{-6} Å^{-2}]	3.7	3.7	3.7
Thickness 2nd Layer*<br>[Å]	20	Thickness 2nd and 4th Layer*<br>[Å]	7	7	7
Thickness 2nd Layer PD	8.1<br>(0.2)	SLD 3rd Layer*<br>[10^{-6} Å^{-2}]	-0.193	-0.193	-0.193
SLD 3rd Layer*<br>[10^{-6} Å^{-2}]	-0.193	Thickness 3rd Layer<br>[Å]	31.6<br>(0.3)	30.53<br>(0.07)	33.84<br>(0.09)
Thickness 3rd Layer*<br>[Å]	42				
SLD 4th Layer*<br>[10^{-6} Å^{-2}]	6.398				
Thickness 4th Layer*<br>[Å]	20				
Thickness 4th Layer PD	4.7<br>(0.1)				
SLD 5th Layer*<br>[10^{-6} Å^{-2}]	-0.193				
Thickness 5th Layer*<br>[Å]	42				
SLD 6th Layer*<br>[10^{-6} Å^{-2}]	6.398				
Thickness 6th Layer*<br>[Å]	20				

Thickness 6th Layer PD	1.27 (0.01)
SLD 7th Layer* [10^{-6} Å^{-2}]	-0.193
Thickness 7th Layer* [Å]	42

Table S2. SASView fitting parameters for bare liposomes (χ^2 = 77.9), PEG-coated liposomes (χ^2 = 431), PSAR-coated liposomes (χ^2 = 356), and PCB-coated liposomes (χ^2 = 11.5) after US insonation. *Parameters fixed during fitting. [a,b]Parameters constrained to equal values during fitting. Errors are reported in parentheses only for fitted parameters.

Parameter	Base	Parameter	PEG	PSAR	PCB
Scale	0.0069 (0.00005)	Scale	0.0554 (0.0004)	0.0683 (0.0005)	0.0460 (0.0002)
Background [cm^{-1}]	0.0557 (0.0003)	Background [cm^{-1}]	0.067 (0.005)	0.0691 (0.0005)	0.07619 (0.0004)
Core & Solvent SLD* [10^{-6} Å^{-2}]	6.398	Core & Solvent SLD* [10^{-6} Å^{-2}]	6.398	6.398	6.398
Core Radius* [Å]	20.0	Core Radius [Å]	555 (2)	442 (7)	416 (1.2)
Core Radius PD	12.2 (0.1)	Core Radius PD	0.069 (0.003)	0.18 (0.01)	0.49 (0.01)
SLD 1st Layer* [10^{-6} Å^{-2}]	-0.193	SLD 1st and 5th Layer[a] [10^{-6} Å^{-2}]	5.38 (0.02)	6.176 (0.002)	6.308 (0.002)
Thickness 1st Layer* [Å]	42	Thickness 1st and 5th Layer[b] [Å]	26.8 (0.2)	100.8 (0.3)	91.0 (1.1)
SLD 2nd Layer* [10^{-6} Å^{-2}]	6.398	SLD 2nd and 4th Layer* [10^{-6} Å^{-2}]	3.7	3.7	3.7
Thickness 2nd Layer* [Å]	20	Thickness 2nd and 4th Layer* [Å]	7	7	7
Thickness 2nd Layer PD	9.6 (0.2)	SLD 3rd Layer* [10^{-6} Å^{-2}]	-0.193	-0.193	-0.193
SLD 3rd Layer* [10^{-6} Å^{-2}]	-0.193	Thickness 3rd Layer [Å]	30.8 (0.3)	30.51 (0.07)	35.1 (0.1)
Thickness 3rd Layer* [Å]	42				
SLD 4th Layer* [10^{-6} Å^{-2}]	6.398				
Thickness 4th Layer* [Å]	20				
Thickness 4th Layer PD	4.9 (0.1)				
SLD 5th Layer* [10^{-6} Å^{-2}]	-0.193				
Thickness 5th Layer* [Å]	42				
SLD 6th Layer* [10^{-6} Å^{-2}]	6.398				
Thickness 6th Layer* [Å]	20				

Thickness 6th Layer PD	1.30 (0.01)
SLD 7th Layer* [10^{-6} Å^{-2}]	-0.193
Thickness 7th Layer* [Å]	42

SUPPORTING INFORMATION REFERENCES

(1) Nele, V.; Holme, M. N.; Kauscher, U.; Thomas, M. R.; Doutch, J. J.; Stevens, M. M. Effect of Formulation Method, Lipid Composition, and PEGylation on Vesicle Lamellarity: A Small-Angle Neutron Scattering Study. *Langmuir* **2019**, *35* (18), 6064–6074. https://doi.org/10.1021/acs.langmuir.8b04256.

(2) Heller, W. T. Small-Angle Neutron Scattering for Studying Lipid Bilayer Membranes. *Biomolecules* **2022**, *12* (11), 1591. https://doi.org/10.3390/biom12111591.

(3) Higaki, Y.; Furusawa, R.; Otsu, T.; Yamada, N. L. Zwitterionic Poly(Carboxybetaine) Brush/Albumin Conjugate Films: Structure and Lubricity. *Langmuir* **2022**, *38* (30), 9278–9284. https://doi.org/10.1021/acs.langmuir.2c01040.
